# Addressing Older Adults' Sexual Health Needs in Regional Primary Care: A Qualitative Study of Barriers and Enablers

**DOI:** 10.1111/ajr.70183

**Published:** 2026-04-02

**Authors:** Louise Bourchier, Lauren Ware, Meredith Temple‐Smith, Jane S. Hocking, Jane Tomnay, Sue Malta

**Affiliations:** ^1^ Sexual Health Unit, Melbourne School of Population and Global Health The University of Melbourne Melbourne Australia; ^2^ Department of General Practice and Primary Care The University of Melbourne Melbourne Australia; ^3^ Centre for Excellence in Rural Sexual Health The University of Melbourne Melbourne Australia

**Keywords:** ageing, health assessment, health promotion, older adults, primary care, regional, rural, sexual health, telehealth

## Abstract

**Objective:**

To explore whether sexual health is discussed in regional settings between primary care clinicians (General Practitioners (GPs) and Practice Nurses (PNs)) and older patients (aged 60 and over), and the barriers and enablers of sexual healthcare for older adults in regional areas.

**Setting:**

Participants were based throughout regional Victoria, with all five regions of Victoria represented.

**Participants:**

Two participant groups were included: older adults aged 60 and over, and primary care clinicians (GPs and PNs). Nine older adults (5 women, 4 men, ranging in age from 60 to 87 years), and 8 clinicians (4 women, 4 men, 6 GPs and 2 PNs) were interviewed.

**Design:**

Data were collected via semi‐structured interviews between July and December 2024. Data were analysed using qualitative content analysis.

**Results:**

Sexual health conversations were rare and ad‐hoc between older adults and primary care clinicians. Key barriers for both groups included stigma around sexual activity and ageing, privacy concerns in small communities, high staff turnover, and structural challenges such as cost and time. Key enablers for both groups included telehealth, systematic inclusion of sexual health in routine care such as the 75+ health check, training for regional clinicians, and health promotion campaigns for older adults living regionally.

**Conclusion:**

Older adults and primary care clinicians agree that sexual health is important to older adults' quality of life, although such conversations are rarely prioritised. This study offers suggestions for clinicians to broach sexual health so that it stays on the agenda as part of older patients' overall care within regional primary care.

## Summary

- What is already known on this subject.
  ○ Sexual health is important to older adults, but it is not often addressed in primary care due to embarrassment, limited time, and precedence of other health issues.
  ○ Australian older adults want to discuss sexual health with healthcare providers, and GPs are willing to have these conversations; however, it is unclear whose responsibility it is to initiate them.
  ○ People in regional areas often encounter additional challenges accessing sexual healthcare, yet no studies to date have explored sexual healthcare access for older populations living regionally.
- What this paper adds
  ○ This paper includes the perspectives of both older adults and primary care clinicians (GPs and Practice Nurses) on the barriers and enablers to sexual health conversations in the regional primary care setting.
  ○ Regional‐specific challenges were identified by both groups, including stigma and conservatism regarding sexual activity and ageing, and privacy concerns in a small community.
  ○ Both groups identified practical ways to support sexual health conversations, including telehealth, including sexual health in routine care such as the 75+ health check, training for regional clinicians, and health promotion for older adults living in regional areas.

## 1 Introduction

In Australia, 1.4 million people aged 65 and over live in regional areas, representing one third of this age group [1]. The Modified Monash Model classifies geographical areas ranging from urban to remote [2], with regional areas between these two extremes representing those living rurally and in smaller centres. While there may be benefits to living in smaller communities, such as close social connections, proximity to nature, and lower housing costs [3], there may also be challenges, including longer travel times to access services, fewer healthcare providers, and limited aged care options [4]. If we are to meet the health needs of older adults living in regional areas, and achieve the UN goal of improving wellbeing and “adding life to years”, as outlined in the Decade of Healthy Ageing [5], it is crucial that we give attention to the specific health needs of older adults living regionally, which may be different from their metropolitan counterparts.

Employing the UN working definition, ‘sexual health’ can be understood as “…not merely the absence of disease… [and] implies that people are able to have a satisfying and safe sex life…” [6]. As a component of overall wellbeing, older adults' sexual health has received increasing research attention in recent years [7, 8, 9]. However, it remains an aspect of healthy ageing that tends to be overlooked for individuals and populations, despite evidence that many older adults' continue to engage in sexual activity and value sexual expression as an important part of their overall wellbeing [10, 11, 12]. Older adults have high engagement with primary care as chronic conditions increase with age [13] and typically see a General Practitioner (GP) 10 or more times a year [13]. When older adults seek sexual healthcare, they usually do so from a GP [14]. Although GPs are willing to discuss sexual health with their older patients, they are unlikely to broach the subject unprompted [15, 16, 17]. Compounding this, many older adults who would like to discuss sexual health with a GP or other healthcare provider do not raise the subject either [16, 17, 18]. Issues such as embarrassment, insufficient time, and more urgent health issues, lead to this stalemate with neither patient nor GP broaching sexual health and it slipping off the agenda [16, 17, 18, 19, 20]. In addition to GPs, Practice Nurses (PNs) play an important role in the primary care setting, providing a range of healthcare including vaccination, wound care, health assessments, education, screening, and the provision of sexual healthcare [21]. Primary care clinicians have been encouraged to initiate sexual health conversations with older patients, especially when related to chronic conditions or medications, and to create an open environment in which older patients will be less hesitant to broach sexual concerns themselves [7].

In the Australian context, the Sex, Age and Me study (2015) provided key information about the sexual health needs and behaviours of older Australians, identifying that many were sexually active, that STI testing was low among those potentially at risk of an STI despite good safer‐sex knowledge, and that sexual health conversations were rare and often awkward with healthcare providers [14, 18, 22, 23, 24]. In addition, the Australian Study of Health and Relationships (2013) highlighted lower sexual health knowledge and condom use among older adults as compared to younger age groups [25]. However, neither of these studies disaggregated data by geographical remoteness, providing no insight into the differences between those ageing in the regions as compared to urban older adults. The Sexual Health Ageing Perspectives and Education (SHAPE) project (2017), which identified barriers to sexual health conversations between GPs and older patients in Victoria, proposed a checklist tool to facilitate these discussions [16, 17, 26]. Building on this, in 2021, we conducted the national SHAPE2 survey exploring the sexual health information‐seeking behaviours and preferences of older adults in Australia [27]. We identified that many older adults do seek sexual health information (41.2%), that older men are more likely to have done so than older women (51.5% compared to 30.6%), and that older adults primarily seek sexual health information from a GP or other healthcare provider (74.1%), followed by searching online (42.6%) [28]. We also found that older adults living in regional and remote areas are less likely to have sought sexual health information than those living in urban centres (37.3% compared to 43.3%) [28].

There is a recognised disparity in healthcare access between those living in urban areas and those living in regional and remote areas in Australia [4]. Residents of regional areas are frequently faced with longer travel distances to access services, especially specialists, as well as longer wait times for appointments, fewer choices of healthcare providers, and often greater cost [29, 30, 31]. In terms of sexual health, the 2023 Australian Senate report ‘Ending the Postcode Lottery’ documented the significant disparities and challenges in sexual healthcare access faced by those living in regional areas [32]. The report identified barriers to accessing contraception, abortion, and maternity care for those living in regional and remote locations, recommending staff training, changes to funding models, as well as an expansion of telehealth [32]. Given that sexual health access challenges are known to exist for regional communities, and for older adults in particular, it seems likely that older adults in regional areas may face additional challenges accessing sexual healthcare.

In this paper we describe the SHAPE3 study, a qualitative study that builds on the earlier sexual health and ageing research of the SHAPE and SHAPE2 projects by turning the focus on the regional context. SHAPE3 was conducted in regional Victoria in 2024 and was designed to explore whether older adults located in regional areas discuss sexual health with their primary care clinicians and the barriers and facilitators to doing so. This research will contribute to our understanding of how the combination of being an older person and living regionally can affect access to sexual healthcare in primary care. The findings of this study can be used to inform policy and services to improve sexual healthcare access for older adults living in regional areas.

## 2 Methods

This study is situated within a constructivist paradigm [33], using qualitative semi‐structured interviews to understand and explore participants' perspectives. Methods of this study are outlined according to the COREQ guidelines for reporting qualitative research [34].

### 2.1 Data Collection

Semi‐structured interviews were conducted with two participant groups: older adults and clinicians. Interviews took place between July and December 2024, either online via Zoom, by phone, or in person and were audio recorded. Interview questions explored whether/how sexual health conversations occur in regional primary care, barriers to these conversations, as well as enablers (see Table A1 in Appendix 1 for interview schedule).

Older adults were eligible if they were aged 60 and over and living in regional Victoria. They were recruited via paid Facebook advertising, emails to local community groups, and through contacting participants from the 2021 SHAPE2 survey who had indicated they were willing to be contacted about future research. Interviews were approximately 50 min in duration.

Clinicians were eligible if they were either GPs or PNs working in regional Victoria in a primary care clinic. They were recruited via emails sent directly to eligible GP clinics and through professional networks. Interviews were approximately 30 min in duration.

All interviews were conducted by LB, a middle‐aged, female PhD candidate holding a Master of Public Health and with a professional background in adult sexual health education.

### 2.2 Ethics and Funding

This study was supported by an R.M. Gibson Research Trust grant from the Australian Association of Gerontology (no grant number). Ethical approval was obtained from the University of Melbourne Human Ethics Committee (ID: 2024‐28248). A 12‐member advisory group comprised of health professionals and older adult community members offered high‐level guidance on the project via two online meetings (one before and one after data collection).

All participants were provided a Plain Language Statement and had the opportunity to ask questions before agreeing to the interview. Written consent was obtained prior to the start of interviews. Each participant received an e‐gift card for their participation in the study. Any identifying information shared in interviews was anonymised to ensure participant confidentiality. All data and participant information were stored in a password‐secured university drive only accessible to the researchers.

### 2.3 Data Analysis

Audio files were transcribed automatically using Otter.ai software and these transcripts were manually cleaned by L.B. and L.W. to ensure accuracy. Transcripts were analysed using qualitative content analysis, a descriptive method commonly used in health research [35, 36], which seeks to summarise and categorise data to accurately communicate the views and experiences of participants without attempting further abstraction. NVivo 15 software was used to facilitate analysis. A mixed deductive‐inductive approach to analysis was used, with results presented in domains of inquiry. LB analysed the whole dataset, LW analysed half the dataset, then both worked together to resolve disagreements and refine the analysis. LB then reviewed the whole dataset again, making further minor adjustments. SM then reviewed a sub‐set of the data, verifying the analysis and contributing final refinements. Participants were not involved in the verification of transcripts or analysis of data.

## 3 Results

### 3.1 Sample

Seventeen interviews were conducted: 9 with older adults and 8 with clinicians. Among older adults, there was a near balance between men (four) and women (five). Their ages ranged from 60 to 87 with two‐thirds aged in their 60s; all had a regular GP, and they were located across four of the five Regions of Victoria (Table 1). Among clinicians, there was a balance between men (four) and women (four). There were six GPs and two PNs, and the proportion of patients they saw aged 60 and over ranged from one in three to nine out of ten. Clinicians were located across four of the five Regions of Victoria (Table 1).

**TABLE 1:** Participant demographics.

	Older adults	Clinicians
Gender		
Female	5	4
Male	4	4
Age		
60–69	6	—
70–79	2	—
80–89	1	—
Clinical role		
General Practitioner	—	6
Practice Nurse	—	2
Region of Victoria		
Barwon South West	0	2
Gippsland	3	3
Grampians	1	0
Hume	4	2
Loddon Mallee	1	1

### 3.2 Findings

Data were organised into three domains of inquiry, with data in each domain grouped into categories. An overview of the analysis is presented in Table 2, with expansion and examples provided below. In discussing patient‐clinician dynamics, most participants felt that similar personal attributes between patient and clinician such as a gender match, closer age, and similar cultural background helped build rapport and made it easier to discuss sexual health. As these findings reflect the literature [18, 37, 38] with no apparent differences in the regional context, these data are not presented here. Quotes are identified by participant number (CL indicating clinicians and OA older adults) and some quotes have been truncated and/or edited for readability.

**TABLE 2:** Overview of findings.

Domain of inquiry	Categories within each domain
(1) Sexual health conversations in practice in regional primary care	(1a) Low knowledge and low priority of sexual health (1b) Limited and incidental conversations about sexual health (1c) Ambiguity about responsibility to initiate conversations (1d) Clinical role of GPs and PNs
(2) Influence of the regional context on sexual health conversations	(2a) Stigma and conservative attitudes in small communities (2b) Confidentiality concerns in small communities (2c) Staff turnover and duration of clinical relationship (2d) Structural and pragmatic factors in regional clinics
(3) Ways to support sexual health conversations in regional primary care	(3a) Telehealth to reduce structural and pragmatic barriers (3b) Systematic inclusion of sexual health in routine care (3c) Training for regional clinicians (3d) Health promotion in regional areas to raise awareness

#### 3.2.1 (1) Sexual Health Conversations in Practice in Regional Primary Care

We sought to understand the contexts in which sexual health conversations occurred in regional primary care between older patients and clinicians, and determine who was responsible for initiating such discussions.

##### 3.2.1.1 (1a) Low Knowledge and Low Priority of Sexual Health

Awareness of older adults' sexual health was generally felt to be low among both clinicians and older patients, underpinning why it was often not addressed. Despite clinicians and older adults both agreeing that “sexual health is important to older patients” (CL5), it was not prioritised or well understood. As expressed by one older adult participant:

I think the key barriers would be, as I said, knowledge again. Putting it on the priority list, it's probably not a priority. (OA8)

There was scepticism of GPs' ability to address older adults' sexual health concerns and whether they had the necessary knowledge and training, with one participant stating:

How experienced is this guy on that sort of stuff, you know? (OA6)

Clinicians echoed these concerns, recognising that older adults “may think that the GP doesn't have enough knowledge or skills*”* (CL5). With an acknowledgement that genuine knowledge gaps may exist, as explained by one GP:

I think for us as clinicians, we need to have a better understanding of what sexual health is and what it means for patients. (CL6)

A sense of resignation towards sex and sexual health was apparent among some older patients who, due to their age, may “think that that their body is no longer allowing them to have a certain experience” (CL5), with little prospect of improvement. The mounting health issues that can come with advanced age underpinned this resignation, as explained by both older adults and clinicians:

With all the kinds of health issues you do get when you're over 65, probably not top of your list. (OA8)

In our priorities of care, it's not up there… because we're just inundated with all the usual chronic health diseases and everything else. (CL1)

##### 3.2.1.2 (1b) Limited and Incidental Conversations About Sexual Health

Older adults expressed mixed views on whether they were comfortable discussing sexual health with primary care clinicians. While some felt confident speaking openly, feeling “not embarrassed about sex at all*”* (OA9), the majority of participants expressed discomfort, as exemplified by this participant quote:

I haven't really had much occasion to do it… I would have to choose the GP carefully… I wouldn't feel that comfortable talking to the local GP about it. (OA7)

Aware that older patients were typically reluctant to broach this sensitive topic with them, some clinicians were proactive in broaching sexual health:

I try always to include sexual health questions in my usual chatting about things, and it comes in quite easily really. (CL4)

Other clinicians felt that “probably sexual health comes up in the general practice setting when there's a problem” (CL7) and was rarely broached directly, but was discussed as an off‐shoot of other matters, as explained by this PN:

Breast screening, cervical screen, we ask are they up to date for their bowel screening, all of those things, and within that, that may well be where they raise… they'll also say “I'm in a new relationship”, or maybe “I'm not in a new relationship”. So, it's a roundabout conversation. (CL8)

##### 3.2.1.3 (1c) Ambiguity About Responsibility to Initiate Conversations

Broaching sexual health was felt to be a shared responsibility of both older patients and clinicians in the regional primary care setting, with more responsibility on clinicians. While a few older adults and clinicians felt that “the patient should be asking the question” (OA2) if they had a specific concern, they also expected clinicians to raise sexual health from time to time, acknowledging that “the patients can be hesitant” (CL5). This balance of responsibility was described by one GP who said:

We've always got to acknowledge the patient's agenda, so obviously it's a patient's responsibility to present with their agenda. But equally, we need to look at opportunistic health screening, of which sexual health becomes part of it. (CL3)

##### 3.2.1.4 (1d) Clinical Role of GPs and PNs

Overall, GPs, PNs, and older patients all felt the GP was the preferred clinician with whom to discuss sexual health, however, a PN was also acceptable. GPs were best positioned due to greater privacy in consultation rooms and established clinician‐patient relationships, as expressed by this PN:

We felt that the practice nurse wasn't the best person placed to be talking about sexual health… We felt that patients may not accept the information as well from a Practice Nurse and they would be more likely to discuss it with a GP that they'd have an ongoing relationship with for quite some time… They've got more privacy—practice nurses' got people in and out of a room all the time. (CL7)

Older adults were open to speaking to a nurse, but tended to prefer a GP due to the perception that a GP has more specialised expertise:

I don't think it would worry me either way. Well, I suppose I've got an attitude in the back of my head that the doctor is more qualified to talk about it than a nurse. (OA4)

#### 3.2.2 (2) Influence of the Regional Context on Sexual Health Conversations

Participants identified a range of factors specific to the regional context that had an impact on older adults' ability to access sexual healthcare in general practice. While overall the regional setting was seen to present barriers, enablers were also identified.

##### 3.2.2.1 (2a) Stigma and Conservative Attitudes in Small Communities

Participants described stigma and conservatism in smaller regional communities as barriers to older adults discussing sexual matters with primary care clinicians. This was both internalised stigma for older patients, who questioned whether sexual interest was appropriate at their age, as well as a fear of negative reactions and judgements from clinicians. Participants felt that “country people tend to be a little bit more conservative” and are “less likely to talk about sexual health openly” (OA4) as compared to those in urban areas. Clinicians were, however, attuned to the inhibiting influence of conservative attitudes, as expressed by this GP:

I think probably the rural situation makes it more difficult for older patients, because there is a societal sort of expectation that perhaps you shouldn't be having sex, you shouldn't be enjoying sex, certainly not enjoying sex. (CL4)

##### 3.2.2.2 (2b) Confidentiality Concerns in Small Communities

The regional context also raised privacy concerns for older adults who were wary that “small town gossip can be horrendous*”* and while they trusted the integrity of the clinicians who were “all professional [and] have their oath of confidentiality”, they were uneasy that “people in the waiting room are not bound by those limits*”* (OA5). Intersecting social circles also meant that older patients were wary of speaking to clinicians who socialised within the community, as described by one participant:

My wife and I often mix socially with a couple of doctors. Now it'd be sometimes a bit uncomfortable to think that you sat down with them the day before talking about sexual function, the next day having dinner with them. (OA4)

Clinicians also understood how this lack of anonymity can make it more challenging for older patients to discuss sexual health with them, as expressed by one GP:

I'm sure it's a barrier that they know that they're going to see me at the supermarket or the pub… and their daughter‐in‐law will work on reception and like, there's so many connections. (CL2)

##### 3.2.2.3 (2c) Staff Turnover and Duration of Clinical Relationship

Participants spoke about how “one of the big things is in rural and remote health is the fact that there's so many [transient GPs] you don't see the same GP most of the time” (CL7), describing how “the lack of consistency with one doctor” is “a major issue” (OA7), making sexual health conversations less likely. As one older adult described:

They come out to regional to do their, whatever they have to do, a certain amount of training in a regional town, especially they come from overseas… and they're gone in a year, sort of thing. So, you don't really get enough time to build up that relationship. (OA8)

Clinicians tended to feel that “if I've known them for a long time, it's more likely for them to be comfortable to talk about something like that” (CL6) with older adults agreeing that “patients are far more comfortable with a long‐term doctor” (OA5). This overall preference for established relationships was clear, however, there was also recognition that “on the flip side, someone that has [sic] not met before might actually be more comfortable talking about it because they don't know anything about their background of history” (CL6) and that “a new doctor might open up avenues that you wouldn't have taken with someone who'd been in the clinic a long time” (OA4).

##### 3.2.2.4 (2d) Structural and Pragmatic Factors in Regional Clinics

Participants spoke of overarching issues that have an impact on the ability of older adults' living regionally to access sexual healthcare. These factors pertained to primary healthcare access as a whole but were seen as particularly affecting sexual health as an issue that tends to slip down in priority.

Concern that regional people sometimes “have to travel so far” (OA5) to reach a clinic, that it was “more difficult to get appointments” (OA3), that “you've got limited time*”* (OA6) in a standard consultation, and that “cost definitely would be” (OA4) a barrier for some people all made it more challenging for older adults to seek sexual healthcare. Clinicians recognised how these structural limitations presented barriers for older patients, stating that “you can't get help for your sexual health issue if you can't see a GP, and that's a massive issue where we are*”* (CL2).

In contrast, some felt that healthcare provision was “incredibly good in this country”, lauding that they could “see a doctor for nothing” (OA9). Additionally, one GP described how it was easier for older patients in her regional clinic to get appointments, compared to the urban clinic she previously worked in:

It's easier for them to actually come and see us compared to maybe the metro counterparts… because sometimes we do have a few reserved spots, so we don't get fully booked out. (CL5)

It should be noted that this example is unusual, with waiting times for primary care in regional communities generally longer than in urban centres [4].

#### 3.2.3 (3) Ways to Support Sexual Health Conversations in Regional Primary Care

Participants saw a range of opportunities to improve sexual healthcare for older patients in regional primary care. These included use of telehealth, training for clinicians working in regional areas, actively seeking opportunities to broach sexual health in routine care, and greater health promotion in regional communities.

##### 3.2.3.1 (3a) Telehealth to Reduce Structural and Pragmatic Barriers

Telehealth was discussed as a way to facilitate sexual healthcare for older adults in regional areas, albeit with the caveat that it would not suit everyone. Telehealth was seen to be *“*good for rural people… [because] they're not spending that time traveling into town to do it and then back home again, they're doing it in their own house” (OA1). Another strength of telehealth was that “it'd be easier over the phone, you're not going to be embarrassed because you don't have that sort of face‐to‐face thing*”* (OA4). While a simple phone call was felt to be generally accessible, for online video calls it was recognised that “there would be some people who couldn't access it*”* (CL7) due to poor internet connectivity and limited digital literacy. Nevertheless, it was felt that older patients were “getting more used to telehealth in a variety of contexts*”* (CL2) and that it would be appropriate for sexual health consultations.

##### 3.2.3.2 (3b) Systematic Inclusion of Sexual Health in Routine Care

There were opportunities to build on the incidental and ad‐hoc sexual health conversations that were already occurring within routine care, and for clinicians to proactively “open a door” (OA4), “break down those barriers” (OA3), and “get the ball rolling” (OA7) with talking about sexual health. Clinicians discussed how when an older patient “had a procedure or some medications*”* the clinician should *“ask directly”* (CL6) about any impacts on sexual function, with older adults agreeing that inquiring about sexual function was “an appropriate way forward” (OA3) when medications had side effects that affected sexual functioning. In addition to broaching sexual health during screening for cervical, prostate, and breast cancer, participants also spoke about the annual Medicare funded 75+ health check as an opportunity for clinicians to raise sexual health with their older patients:

I don't think Medicare mandates sexual health as a discussion point, and if that was incorporated into the 75‐year‐old health check, that's a way in which it can be combined with screening. (CL3)

A pre‐consultation checklist for older patients to identify issues they would like to talk about with their GP, was also seen as “*very practical*” (OA6) and “a good idea” (OA7) to prompt sexual health conversations.

##### 3.2.3.3 (3c) Training for Regional Clinicians

Older adults emphasised the importance of having a clinician who was “on top of their job” (OA4) and described how “educating doctors is number one*”* (OA8) to get sexual health on the agenda. Clinicians recognised that “the way that we were taught sexual health at university, (a) was basically not at all, and (b) was like venereal diseases, and not much else” (CL2) and that additional training may be needed in older adults' sexual health. They offered suggestions for professional development to upskill regional clinicians that could be provided or accredited by RACGP and other relevant bodies. For example, one GP spoke about using an audit‐based training method that could be applied to older adults' sexual health, as he described:

As part of the audit, you have to think about your last five consultations and take “yes” or “no”, did you actually ask the question? And if not, what were the barriers of doing so, and so that can be a prompt… something like that [on sexual health and ageing] can be designed as part of the RACGP curriculum or CPD module. (CL6)

##### 3.2.3.4 (3d) Health Promotion in Regional Areas to Raise Awareness

Participants saw community health promotion and education as important to reduce stigma and increase knowledge of sexual health among older populations. Key messages were that sexual health is “okay to talk about*”* (CL8) with a GP and “that it's not something you'd have to come to a specialist*”* (OA2). To raise awareness, they suggested “public awareness talks” (CL1), that it would be good “if the community health had a session on sexual health for people over 65 every now and then” (OA1), and “if there was more information talks available to seniors' groups*”* (OA6). Posters and brochures could also be placed in clinic waiting rooms, with participants saying “I'm a great believer in posters in the waiting room” (CL4) and that “it would be a positive thing, I think, to have signs up” (OA4). However, while some said they “would probably go and pick up the brochure” (OA9) they also acknowledged that people may “feel embarrassed if somebody sees them picking up a brochure about a sexual health problem” (OA5), and that posters and brochures may “lose their impact over time” (CL1) and be a “waste of time” (OA6). They suggested instead that “social media now is the way to go” (OA6) and that any health promotion campaign needed to be “*multi‐factorial*” including “the internet, the digital media, TV, newspapers, all those things” (OA7).

## 4 Discussion

In this qualitative study, we found a range of factors that influence older adults' access to sexual healthcare within regional primary care. Through interviews with 17 participants in regional Victoria, Australia, we were able to explore the views of primary care clinicians and older adults to understand the specific impact of the regional setting on sexual healthcare access for older patients and found a high degree of congruence between both groups. Study findings are discussed and explored below, culminating in recommendations to support the sexual health of regional older adults going forward.

Studies exploring the management of older adults' sexual health in primary care, in Australia as well as overseas, have found that clinicians are willing to discuss sexual health, and that older patients would like the opportunity to do so, but that these conversations remain rare due to embarrassment, time constraints, and precedence of more urgent health concerns [17, 18, 19, 20, 26]. We found that these same dynamics are at play in the regional Victorian context. Our research also aligned with the literature regarding regional healthcare in Australia, identifying the challenges faced by many who live regionally, including longer travel distances, fewer choices of healthcare providers, cost, and disruptions to continuity of care due to high staff turnover [29, 30, 31]. These barriers, unsurprisingly, also affect sexual health, potentially more so by relegating it to a low priority issue. The 2023 report “Ending the Postcode Lottery” highlighted inequitable access to sexual and reproductive healthcare for those living in regional areas, citing long travel distances to specialists and variability of primary care clinicians in addressing sexual health [32]. While some issues, such as abortion and contraception, are less relevant to older age groups, the pattern of access to sexual healthcare appears the same. Our research found that older adults in regional settings may delay seeking sexual healthcare due to privacy concerns in local clinics where staff and other patients may be known to them. These concerns about confidentiality are known to hinder sexual healthcare seeking among regional young people [32, 39], and the findings highlight how these concerns persist for older age groups. Our participants described how conservative attitudes in smaller communities, including discomfort with (and in some cases disapproval of) sexual activity among older adults, amplified the barriers for those ageing in the regions. This research brings to the foreground the compounding barriers at the nexus of regional location, sex, and ageism.

As real as these challenges are, our research also captured positives that enable older adults' sexual healthcare access in the regional context. The study findings indicate the willingness and motivation of clinicians working regionally to address older patients' sexual health, an issue they know to be important. Structural barriers such as limited time and high staff turnover inhibit this from being achieved; however, participants identified opportunities to expand telehealth and include sexual health in the 75+ health check to mitigate these challenges.

Telehealth is available for sexual health consultations, with specific Medicare item billing numbers allowing sexual health appointments with a GP who is not a patient's regular GP, or with a specialised sexual health service, and there are also options for free ‘bulk billed’ sexual health telehealth services. Awareness of telehealth services, and how to access them, is key to improving equitable access to sexual health services for people living regionally, including older adults, so that they can make use of these available options. While study participants identified limitations to the use of telehealth, they saw opportunities to upscale it to support the sexual health of some rural older adults.

The 75+ health check is a comprehensive annual health assessment offered to patients aged 75 and over. Currently the assessment does not include sexual health. A comprehensive health assessment presents an opportunity to ask about sexual health periodically as part of overall health, and our participants saw this as an acceptable way to raise the topic. Formalising the inclusion of sexual health in the 75+ health check would add structure to the current ad‐hoc approaches of broaching sexual health with older patients and was encouraged by both participant groups. Additionally, the finding that a pre‐consultation checklist is acceptable to older patients to prompt sexual health conversations is consistent with the findings of the earlier SHAPE study [26].

Increasing clinician knowledge and awareness is also a key piece of the puzzle and one that would require only moderate resourcing to see benefits. As regional clinicians are already including sexual health in routine care to some extent, offering further training with specific strategies for when and how to broach sexual health with older patients would reinforce these existing approaches.

A tentative but novel finding of this study is that GPs, rather than PNs, may be the preferred clinician to address older adults' sexual health in regional primary care, with both patients and clinicians tending to agree with this. Enabling nurses to undertake a wider range of consultations and procedures has generally been advocated to improve sexual health access in regional areas, with some regional clinics moving to nurse‐led models of practice [40, 41]. However, the complexity of comorbidities, privacy concerns, and less established relationships with PNs than with GPs may mean that, while nurse‐led consultations are suitable for younger people, they may not be preferable for older patients in regional settings. These findings should be treated as preliminary pending further research on this question.

Community health promotion campaigns are also important to raise awareness and normalise sexual health among older adults. These initiatives should address concerns around privacy, stigma, and reassure older patients that their GP is willing and able to discuss sexual health. Our participants saw a role for educational talks for community groups such as the University of the Third Age and Rotary, as well as education sessions and awareness campaigns through local health services that already offer programming for older citizens.

### 4.1 Recommendations

To bring these findings together and identify ways they can be put into practice to improve sexual healthcare for older adults in regional areas, we outline the following five recommendations:

1. Regional GPs are encouraged to proactively ask older patients about sexual health from time to time as part of routine preventive care. Sexual health should be raised specifically when health conditions or medications may have sexual impacts, as well as during screening of adjacent health issues such as prostate and cervical screening. Hinchliff et al. [7] outline guidelines for GPs in how to create an environment conducive to sexual health conversations, and how to broach and navigate them. This recommendation is based on finding 3b and informed by findings 1b and 1c.
2. Training in sexual health and ageing should be offered to regional GPs. Peak bodies such as the Royal Australian College of General Practitioners (RACGP), the Australian College of Rural and Remote Medicine (ACRRM), the Centre for Excellence in Rural Sexual Health (CERSH) and other relevant organisations should offer and/or accredit relevant training to primary care clinicians to increase knowledge, skills and confidence in addressing the sexual health concerns of older patients. This recommendation is based on finding 3c and informed by finding 1a.
3. The 75+ health check should include sexual health. This Medicare rebated comprehensive annual health check for those aged 75 and over provides a key opportunity to identify sexual health concerns. Sexual health is not currently included in the schedule of questions and its inclusion is recommended. This recommendation is based on finding 3b and informed by findings 1b and 1c.
4. Telehealth for sexual health should be promoted. There are telehealth Medicare item numbers for sexual health, and patients are able to book appointments with a GP who is not their regular GP, including sexual health specific services. These services should be promoted to increase awareness and uptake among older adults in regional settings. This recommendation is based on finding 3a and informed by findings 2a, 2b and 2d.
5. Health promotion for regional older adults should include sexual health. Local health organisations and community groups should include sexual health within their programming for older adults. This health promotion should reassure older adults that they can speak to their GP about sexual concerns and should emphasise that consultations are private and that their primary care clinician is non‐judgemental. This recommendation is based on finding 3d and informed by finding 1a.

### 4.2 Strengths and Limitations

Opt‐in bias must be acknowledged, as non‐random sampling meant that participants who had a prior interest in sexual health and greater comfort talking about it were likely overrepresented. A further limitation is that few NPs were included in the sample despite efforts to recruit more, thus we heard from more GPs than NPs when capturing clinician perspectives. While a small number of participants were of culturally and linguistically diverse backgrounds, overall, the sample was comprised mainly of Caucasian individuals.

There are a number of strengths of this study to highlight. Firstly, there was a gender balance between male and female participants. Secondly, two stakeholder groups were included: both clinicians and older adults, providing perspectives of both healthcare professionals and older patients. Thirdly, this exploratory study is one of very few addressing the sexual health of older adults in the regional context, underscoring the need to shine a light on this often‐overlooked issue.

## 5 Conclusion

This study investigated the unique challenges rural older adults may experience in accessing sexual healthcare, examining the nexus of barriers that an older age and a rural postcode may present. We found that sexual health is important to older adults living in regional areas, and that this is recognised by their primary care clinicians. However, currently, sexual health conversations between older adults and primary care clinicians are occasional and ad‐hoc in nature. Building on this foundation, there are opportunities to improve sexual healthcare through clinician education, community health promotion, upscaling telehealth, and including sexual health within the 75+ health check. These measures have the potential to increase sexual healthcare equity for older adults living in regional areas in Australia and contribute to the goal of improving the wellbeing of older citizens in line with the UN Decade of Healthy Ageing [5]. This study offers practical ways to address older adults' sexual health needs in regional primary care and to get sexual health on the agenda regardless of a patient's age.

## Author Contributions

**Louise Bourchier:** conceptualization, funding acquisition, methodology, investigation, project administration, data curation, formal analysis, writing – original draft, writing – review and editing. **Lauren Ware:** data curation, formal analysis, writing – original draft, writing – review and editing. **Meredith Temple‐Smith:** conceptualization, methodology, writing – review and editing, supervision. **Jane S. Hocking:** conceptualization, methodology, writing – review and editing, supervision. **Jane Tomnay:** conceptualization, methodology, writing – review and editing. **Sue Malta:** conceptualization, methodology, formal analysis, writing – review and editing, supervision.

## Funding

This study was supported by an R.M. Gibson Research Trust grant from the Australian Association of Gerontology (no grant number). J.S.H. was supported by a NHMRC Investigator Grant (2025960). L.B. was supported by an Australian Government Research Training Program scholarship.

## Disclosure

No generative AI tools were used in the preparation of this manuscript.

## Ethics Statement

Ethical approval was obtained from the University of Melbourne Human Ethics Committee (ID: 2024‐28248).

## Conflicts of Interest

The authors declare no conflicts of interest.

## Data Availability

The data that support the findings of this study are available on request from the corresponding author. The data are not publicly available due to privacy or ethical restrictions.

## Appendix 1

**TABLE A1:** Interview schedule.

	Older adults	Clinicians
General background	Community of residence Engagement with healthcare Satisfaction with local healthcare	Community GP practice is in Patient population
Sexual health conversations	Meaning and importance of sexual health Whether/how they and their peers would discuss sexual health in primary care Patient and clinician roles in sexual health conversations Barriers to sexual health conversations Specifics of the rural context	Meaning and importance of sexual health Whether sexual health is discussed with older patients Patient and clinician roles in sexual health conversations Barriers to sexual health conversations Specifics of the rural context
Best practice	Best‐case scenario for older adults seeking sexual healthcare in primary care Ways to raise awareness of sexual health Ways for sexual health to be raised in primary care consultations Role of telehealth	Best‐case scenario for older adults seeking sexual healthcare in primary care Ways to raise awareness of sexual health Ways for sexual health to be raised in primary care consultations Role of telehealth
